# Genome Sequence of the Fungal Strain 14919 Producing 3-Hydroxy-3-Methylglutaryl–Coenzyme A Reductase Inhibitor FR901512

**DOI:** 10.1128/genomeA.00129-17

**Published:** 2017-04-06

**Authors:** Hiroya Itoh, Makoto Matsui, Toshitaka Kumagai, Masanori Arita, Masayuki Machida, Takashi Shibata

**Affiliations:** a Biotechnology Labs, Astellas Pharma, Inc., Ibaraki, Japan; b Technology Research Association of Highly Efficient Gene Design (TRAHED), Hokkaido, Japan; c Fermlab, Inc., Tokyo, Japan; d National Institute of Genetics, Shizuoka, Japan; e Bioproduction Research Institute, National Institute of Advanced Industrial Science and Technology (AIST), Sapporo, Hokkaido, Japan

## Abstract

Fungal strain 14919 was originally isolated from a soil sample collected at Mt. Kiyosumi, Chiba Prefecture, Japan. It produces FR901512, a potent and strong 3-hydroxy-3-methylglutaryl–coenzyme A (HMG-CoA) reductase inhibitor. The genome sequence of fungal strain 14919 was determined and annotated to improve the productivity of FR901512.

## GENOME ANNOUNCEMENT

Originally isolated from a soil sample collected at Mt. Kiyosumi (Chiba Prefecture, Japan), a sterile fungal strain, 14919, produces 3-hydroxy-3-methylglutaryl–coenzyme A (HMG-CoA) reductase inhibitor called FR901512 (1). This compound shares structural similarity with mevastatin and lovastatin, but its core structure is tetralin instead of hexahydro-naphthalene as in the natural statins. FR901512 inhibited cholesterol synthesis from [^14^C]acetate with a 50% inhibitory concentration (IC_50_) of 1.0 nM in the human hepatoma cell line (Hep G2 cells), and it also inhibited HMG-CoA reductase activity with an IC_50_ of 0.95 nM (1). A single oral administration of FR901512 strongly inhibited sterol synthesis in rats, and the once-daily oral administration to beagle dogs decreased their plasma cholesterol level (2).

As the producer of this potent and strong hypolipidemic drug, we undertook whole-genome sequencing of the fungal strain 14919. From its liquid culture for 4 days in potato dextrose broth at 25°C with 180 rpm, cells were protoplasted, and the DNA was purified by NucleoBond AXG 100.

Purified DNA was sequenced on the PacBio RSII single-molecule real-time (SMRT) method (chemistry version P6-C4). A total of 703.8 Mbp in 62,301 PacBio subreads were obtained from 1 cell of the single-molecule real-time (SMRT) cell. The prepared DNA was also sequenced using the Illumina MiSeq platform with the TruSeq DNA LT sample prep kit to obtain 21.3 M paired-end (insertion length, 572 bp; total 12.6 Gbp) short-read data. The draft genome sequence was assembled by MaSuRCA assembler version 3.2.1_12132016 (3). The resulting assembly had 104 scaffolds, with an *N*_50_ value of 711,440 bp. The total length of assembled genome sequence was 48.7 Mbp.

In addition, RNA sequencing (RNA-Seq) was carried out in production medium. RNA extraction and library preparation were done with the TruSeq standard mRNA LT sample prep kit and MiSeq reagent kit version 3 (150 cycles). A total of 72.9 M paired-end (total, 5.5 Gbp) RNA-Seq reads were sequenced by the Illumina MiSeq platform and assembled using Cufflinks version 2.2.1 (4).

A total of 14,342 coding sequences (CDSs) were predicted using CodingQuarry version 2.0 from the draft genome sequence, together with assembled transcripts (5). tRNA genes were predicted by tRNAscan-SE version 1.23 (6). The 5S and 18S-5.8S-26S rRNA genes were identified by a similarity search. The identified 5.8S rRNA gene and internal transcribed spacers 1 and 2 (ITS1 and ITS2, respectively) sequences shared 100% identity with *Xylaria grammica*, an endophytic fungus causing white wood rot.

A polyketide synthase (PKS) cluster of eight genes was considered responsible for FR901512 biosynthesis, of which all genes showed similarity with the lovastatin biosynthetic genes in *Aspergillus terreus*. The novelty of this cluster-wide similarity will be presented elsewhere.

### Accession number(s).

This whole-genome shotgun project has been deposited at DDBJ/EMBL/GenBank under the accession no. BDMC01000001 to BDMC01000104. The version described in this paper is the first version.
